# Patient-profiled treatment responses in a large hypertension trial: a posthoc analysis of the INSIGHT study

**DOI:** 10.1097/HJH.0000000000004284

**Published:** 2026-03-18

**Authors:** Peter W. De Leeuw, Giuseppe Mancia, Chris R. Palmer, Luis M. Ruilope, Solko W. Schalm, Morris J. Brown

**Affiliations:** a Department of Medicine, Maastricht University Medical Center and Cardiovascular Research Institute Maastricht (CARIM), Maastricht, The Netherlands; b University Milano Bicocca, Milan, Italy; c Formerly Centre for Applied Medical Statistics, Department of Public Health and Primary Care, University of Cambridge, UK; d Hospital 12 de Octubre, Madrid, Spain; e Erasmus University Rotterdam and Therapy Selector, Rotterdam, The Netherlands; f Clinical Pharmacology & Precision Medicine, William Harvey Research Institute, Faculty of Medicine and Dentistry, Queen Mary University of London, Charterhouse Square; g Department of Endocrinology, St Bartholomew's Hospital, Barts Health, London, UK

**Keywords:** blood pressure control, co-amilozide, INSIGHT trial, nifedipine, patient-profile

## Abstract

**Objective:**

Guidelines on the treatment of hypertensive patients usually refer to ‘average’ patients. However, in clinical practice, individual patient characteristics may differ substantially from the average. Thus, it seems worthwhile to examine the relationship between comprehensive patient profiles and blood pressure responses.

**Methods:**

We divided the patient population from the INSIGHT trial into exploration and validation cohorts and constructed composite patient profiles based on predictors of blood pressure control (age, severity of hypertension, comorbidities, and previous treatment status). Next, we tested in the exploration cohort whether blood pressure control rates and adverse effects after 6 months of therapy across these profiles differed from those in the entire patient group. Finally, we explored whether the results from the exploration cohort could be validated using another cohort.

**Results:**

Logistic regression analysis showed that the odds of achieving blood pressure control differed substantially between patient profiles but not between treatment modalities. Patients with a less favorable profile (e.g. the combination of age above 60 years, baseline systolic pressure above 160 mmHg, and the presence of diabetes) did less well than patients with a low-risk profile (e.g. absence of organ damage). These results were confirmed in the validation cohort.

**Conclusion:**

We conclude that responses to antihypertensive treatment vary in a clinically important manner depending on the composite patient profiles. When found in other trials as well, a priori knowledge about response rates of various patient-profile treatment regimens may help choose the best treatment in individual patients and improve overall blood pressure control rates.

## INTRODUCTION

Data from the Third National Health and Nutrition Examination Survey have shown that well controlled blood pressure (BP) virtually eliminates the excess risk of hypertension for cardiovascular morbidity and mortality [1]. To achieve adequate BP control, most clinicians base their treatment decisions on the recommendations of the guidelines. Currently, guidelines advocate the initiation of antihypertensive treatment with two drugs rather than one in most patients [2,3]. This is based on evidence that timely control of BP may be associated with a lower incidence of cardiovascular complications, particularly in patients with a high cardiovascular risk [4,5]. Importantly, control rates with any antihypertensive agent were much lower when administered as monotherapy compared to those with dual treatment. However, there are arguments that contradict such a general approach of always starting with two drugs. First, a sizeable proportion of patients still respond very well to monotherapy. It would be unwise to expose such patients to the potential side effects of these two drugs when only one is sufficient. Second, a two-drug regimen is associated with higher costs, which would be less acceptable from a societal perspective if a less costly treatment regimen is sufficient.

Regardless of the choice for mono-therapy or dual-therapy, most physicians do not know which type of patient can be controlled with which medication within a reasonable period. This may be one of the reasons for the disappointing low BP control rates worldwide [6]. In several trials, subgroup analyses were performed to explore the effects of certain medications in relation to the demographic and clinical characteristics of patient populations. These studies have documented the value of factors such as age, presence of diabetes, height of SBP, severity of hypertension, BMI, prior treatment, and others in predicting BP responses to antihypertensive treatment [7]. However, such analyses are usually based on one factor at a time and not on a more complete patient profile. Furthermore, whether these factors are relevant to the occurrence of side effects has not been properly investigated. Having in mind that for the solution of these important questions we could benefit from a more nuanced subgroup analysis [8], we reanalyzed the data from a large antihypertensive treatment trial (the INSIGHT study). We hypothesized that BP responses and side effects in that trial would differ significantly between patient profiles constructed by a combination of individual predictors. To test whether the results would have external validity, we randomly split our patient population into two parts: one for generating the prediction model and the other for testing this model.

## MATERIALS AND METHODS

### Design of the trial

The details of the multicenter INSIGHT trial have been described previously [9]. The inclusion criteria were age 55–80 years, hypertension (BP ≥150 mmHg systolic and ≥95 mmHg diastolic, or ≥160 mmHg systolic regardless of diastolic pressure), and at least one additional cardiovascular risk factor. After 4 weeks of placebo treatment, the patients were randomized to either nifedipine 30 mg daily or co-amilozide (hydrochlorothiazide 25 mg and amiloride 2.5 mg) daily. Patients in whom BP decreased by less than 20/10 mmHg or remained higher than 140/90 mmHg received one of four dose-titration steps: dose doubling of the randomized drug, addition of atenolol 25 mg daily (or enalapril 5 mg daily if atenolol was contraindicated), dose doubling of the additional drug, and addition of any other antihypertensive drug except calcium channel blockers or diuretics.

BP was measured three times after a 5-min rest, with a calibrated mercury sphygmomanometer. After dose titration, patients were observed three times a year for assessment of BP and heart rate.

The primary outcome of the trial was the composite endpoint of cardiovascular death, myocardial infarction, heart failure, and stroke. The study complied with the principles of good clinical practice and the Declaration of Helsinki and was approved by the relevant ethics committee. Written informed consent was obtained from all the patients.

For the present analysis, we only used BP data at baseline and after 6 months of treatment. In addition, we assessed the incidence of side effects at 6 months in relation to patient profiles.

### Construction of patient profiles

Composite patient profiles were constructed based on the following predictors: age, severity of hypertension, comorbidity, and previous treatment status, each of which had several options after transformation into categorical variables (Table 1). We used age as a proxy for the pathophysiological stage of hypertension based on the principle that the activity of the renin-angiotensin system (which progressively falls during life) is a modifier of the response to antihypertensive treatment [10]. The severity of hypertension has four options based on the level of BP and the presence or absence of hypertension-mediated organ damage in the brain, heart, or kidney. Cerebrovascular damage is thought to be present in cases with relevant clinical signs and symptoms. Cardiac damage was diagnosed when objective evidence of left ventricular hypertrophy and/or coronary heart disease was present. We considered an estimated glomerular filtration rate (eGFR) of less than 60 ml/min/1.73 m^2^ or albuminuria without intrinsic renal disease as indicative of renal damage. The last two predictors, comorbidity and previous treatment, had four and two options, respectively, that is, none, obesity, diabetes, and obesity plus diabetes for comorbidity and treatment-naivety or prior therapy with one or more drugs in the last 6 months for previous treatment status. Smoking and cholesterol levels were not included in the profiles, as a previous analysis from our group showed that these risk factors have little, if any, effect on BP responses [11].

**TABLE 1:** Categories of predictor variables

*Age* (1) <60 years
(2) 60–80 years
*Severity of hypertension*
(1) SBP < 160 mmHg, no organ damage
(2) SBP < 160 mmHg and organ damage
(3) SBP ≥ 160 mm Hg, no organ damage
(4) SBP ≥ 160 mmHg and organ damage
*Comorbidity:* (1) None
(2) Obesity (BMI>30 kg/m^2^)
(3) Diabetes
(4) Diabetes and obesity
*Previous treatment status*
(1) Treatment-naive
(2) Previous treatment

Altogether, 2 × 4 × 4 × 2 = 64 composite patient profiles are theoretically possible (see Supplemental file 1); however, to ensure a reasonable estimate of treatment effects, we restricted our analysis to those profiles for which at least 50 observations were available. Individual patients for whom data essential for patient profiling were lacking were excluded from the analysis.

### Calculation of risk scores

As patient profiles can only be analyzed as categorical variables, we also assigned a (continuous) risk score to each profile. To this end, we used a multivariable logistic regression equation that described the relationship between predictors and failure to achieve BP control as the basis for risk calculation. The total score was derived from the sum of the constant and scores for each categorical predictor (see Supplemental File 2 for more details).

### Statistical analysis

Prior to any statistical analyses, we divided our population into two random samples, balanced for treatment, comprising 70 and 30% of the patients. The larger fraction served as the exploration cohort, and the other as the validation cohort.

Using binary logistic regression and the Wald chi-square statistic, we first assessed whether the primary outcome, that pressure control at six months (yes/no), correlated with the individual components that make up the various patient profiles. In this regard, we defined BP control as a SBP of 140 mmHg or less with the drug to which patients were initially assigned. This value was selected because it is the value recommended as the treatment target by most guidelines at the time of the trial [12,13].

For both therapies within the INSIGHT trial, we calculated the ratios of the odds of attaining BP control among the composite patient profiles to those in the entire group. Whenever appropriate, we applied Oldham's correction to the baseline BP to avoid spurious relationships with changes in BP [14,15]. Finally, we assessed the success rates of the various profiles according to their risk scores for both treatments separately. Ordinary linear regression analysis was used to determine the relationship between the risk score and rate of BP control.

For external validation, we applied the results from the exploration cohort to the validation cohort and compared the observed outcome with the expected outcome.

The same kind of analyses were performed with respect to side effects.

Unless otherwise indicated, continuous data are expressed as medians and interquartile ranges, and categorical data are expressed as percentages. Odds ratios are presented with 95% confidence intervals (95% CIs). The Chi-square test was used to compare percentages between the groups. Statistical significance was set at a *P* value less than 0.05.

## RESULTS

Of the 6321 patients who were randomized to treatment, 403 had to be excluded because either BP at 6 months was lacking or because there was not enough information to base one of the prespecified profiles on. The remaining 5918 patients were assigned to 59 of the 64 patient profiles (see Supplemental file 3). However, many of these profiles contained a limited number of patients and only 16 qualified for further analysis because they comprised 50 or more patients.

Table 2 shows the baseline characteristics of the 4809 patients who were included in the statistical analysis. There were no apparent differences between the two treatment groups. The exploration cohort comprised 1687 patients on co-amilozide and 1669 on nifedipine. The validation cohort consisted of 731 and 722 patients, respectively.

**TABLE 2:** Baseline characteristics of the patients included in the analysis

	Co-amilozide (*n* = 2418)	Nifedipine (*n* = 2391)
Age (years)	66 ± 6	66 ± 6
Sex (male/female, %)	46/54	46/54
BMI (kg/m^2^)	27.6 ± 4.2	27.6 ± 4.4
Baseline SBP (mmHg)	174 ± 14	174 ± 14
eGFR < 60 ml/min/m^2^ (*n*, %)	797 (33%)	748 (31%)
Target organ damage (*n*, %)	1239 (51%)	1227 (51%)
Obesity (*n*, %)	564 (23%)	558 (23%)
Diabetes (*n*, %)	399 (16%)	389 (16%)
Treatment-naive (*n*, %)	161 (7%)	157 (7%)

### Exploration cohort

In the exploration cohort, SBP at 6 months had fallen by a median of 32 (IQR 24–42) mmHg in the co-amilozide group and 30 (IQR 22–42) mmHg in the nifedipine group compared to baseline. Altogether, 57% of the patients on co-amilozide and 48% of those on nifedipine reached a SBP below 140 mmHg. This difference was statistically significant (*P* < 0.0001).

In the univariable binary logistic regression analysis, the rate of BP control was inversely correlated with age (*P* = 0.001), severity of hypertension (*P* < 0.001), comorbidity (*P* < 0.001), and previous treatment status (*P* = 0.04). The presence of organ damage contributed significantly to a lower rate of BP control in patients with a baseline SBP of at least 160 mmHg (*P* < 0.001) but not in those with a lower SBP (*P* = 0.15). The correlation with organ damage was driven entirely by the presence of left ventricular hypertrophy (*P* = 0.02), while the presence of obesity (*P* = 0.004), diabetes (*P* < 0.001), or their combination (*P* < 0.001) was a significant predictor of a lesser response in the comorbidity category. In the multivariable logistic regression analysis, all factors except obesity without diabetes remained significant. Although slightly more men than women attained BP control, sex had no significant effect on other factors.

**FIGURE 1:**
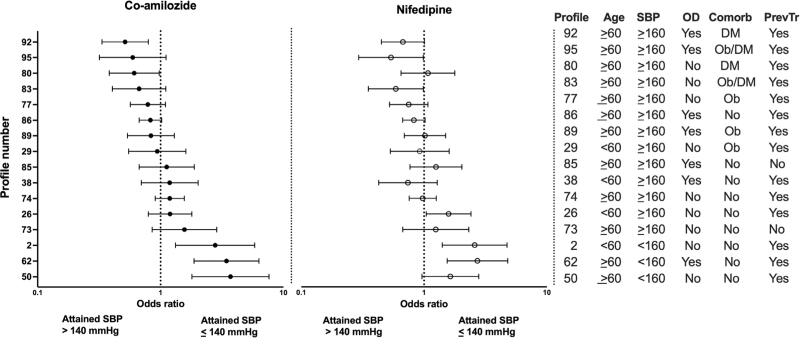
Odds ratios with 95% confidence intervals for achieving blood pressure control (systolic pressure ≤ 140 mmHg) for the various profiles as compared to that in the whole group. Profiles indicated in the table on the right. Comorb, comorbidity; DM, diabetes mellitus; Ob, obesity; OD, organ damage; PrevTr, previous treatment.

Logistic regression analysis further showed that in both the co-amilozide and nifedipine groups, the odds of achieving better or worse than average BP control correlated significantly with the composite patient profiles. Success rates appeared to differ substantially among different patient profiles within the same treatment group; however, for a given profile, they were reasonably similar when the two treatment groups were compared (Fig. 1).

**FIGURE 2:**
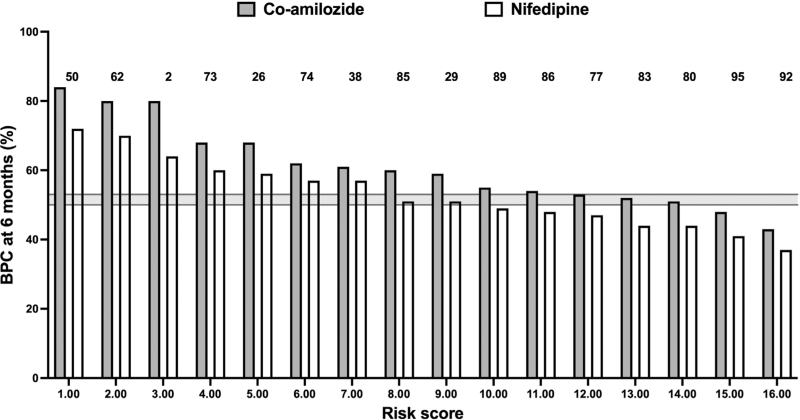
Blood pressure control rates in relation to specific patient profiles for the two treatment regimens. Profiles depicted in ascending order of risk scores. The shaded area represents the overall blood pressure control rate with its 95% confidence intervals. Numbers above the bars refer to the same profiles as in Fig. 1.

Figure 2 shows BP control rates for the various patient profile treatment subgroups in ascending order of their risk scores. The control rates for the different patient profiles varied between 37 and 84% (43–84% for co-amilozide and 37–72% for nifedipine). In both groups, a significant inverse relationship was found between the risk score and BP control (*P* < 0.0001 for both). Although the slopes of the two regression lines did not differ significantly, their intercepts did (*P* < 0.001), with the highest value for the co-amilozide group.

### Validation cohort

**FIGURE 3:**
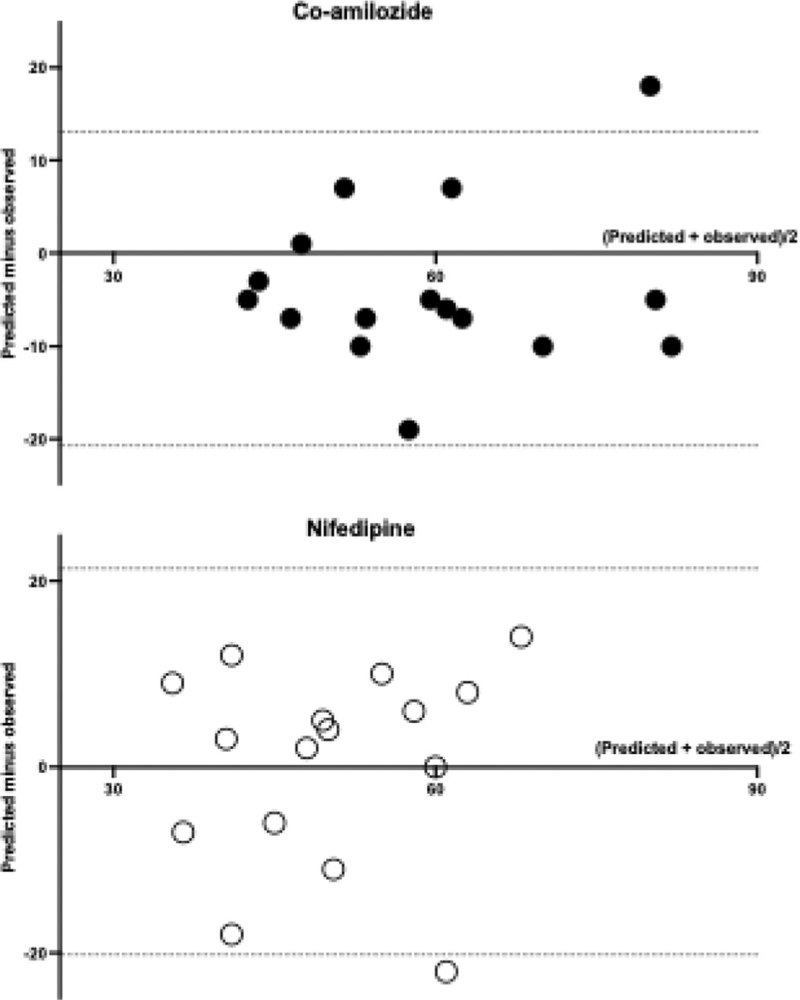
Bland-Altman plots for the predicted versus the observed percentages of blood pressure control in the co-amilozide group (upper panel) and the nifedipine group (lower panel).

In the validation cohort, BP control at 6 months was achieved in 56% of patients in the co-amilozide group and in 50% of those in the nifedipine group (*P* < 0.05). Figure 3 shows the Bland-Altman plots of the predicted (based on the exploration cohort) versus the observed percentages of BP control in the validation cohort. The mean bias was -4% in the co-amilozide group and +1% in the nifedipine group, which did not differ significantly from zero. Likewise, the relationship between the profile risk score and the success rate did not differ between the two cohorts.

#### Side effects

We found no association between any of the patient profiles and occurrence of side effects.

## DISCUSSION

The main finding of the present analysis is that the responses to antihypertensive treatment vary in a clinically important way from the average, depending on patient profiles composed of multiple characteristics. For decades, evidence-based medicine has compared antihypertensive drug treatments by group outcomes, such as BP reductions or events, but the present analysis shows that, at least as far as BP reductions are concerned, differences in outcomes within groups with the same medication may be much larger than differences between treatment groups. By building more comprehensive patient profiles based on a combination of established predictive factors rather than single ones, we were able to identify various profiles that are associated with either better or worse than average BP control. When expressed as a risk profile, the data showed that profiles that represent a more severe phenotype of hypertension (higher BP, more severe hypertension, and comorbid conditions such as obesity and/or diabetes) are associated with more resistance to treatment. In fact, patients with such a phenotype were approximately twice as likely to achieve BP control than patients with a SBP not exceeding 160 mmHg and without any additional abnormalities. Remarkably, these results were similar between the two treatment groups. Nevertheless, it should be emphasized that even though the predictions that came out of the exploration cohort performed as well in the validation cohort, it remains to be established whether our results can predict outcomes equally well in different populations with other types of treatment.

The present results add to the existing knowledge in that a more comprehensive patient profile was evaluated. As we prespecified the profiles before analyzing the data, and because we compared the various profiles with the overall treatment effect, we tried to avoid as much as possible the risks associated with subgroup analyses [16].

Although we did not find major differences between the two treatments used in this study, the effects of co-amilozide tended to be slightly better than those of nifedipine in almost all patients and risk profiles. This finding helps to separate true biological variability from random statistical fluctuation, one of the caveats of subgroup analysis, and strongly suggests that irrespective of the two drugs, the patient profile was the main determinant of the fall in pressure. The fact that we did not find any association between the occurrence of side effects and the various profile groups also argues against random fluctuations as an explanation for our BP findings.

One practical implication of our data is that the use of composite patient profiles may predict the expected response from various therapies for an individual patient. This information may help physicians achieve better BP control rates. In patients with a low-risk profile, it is likely to start treatment with a single drug without obvious prejudice for the possibility of achieving timely BP control. First-step monotherapy in low-risk individuals may also represent an advantage for patient safety because there is evidence that the risk of serious side effects leading to treatment discontinuation increases progressively with the number of prescribed drugs [17–19]. A second implication of our findings is that, in contrast, patients with organ damage and/or comorbidities (obesity and diabetes) are better off when they start immediately with a two-drug combination and are candidates for three-drug combination therapy.

One of the limitations of our study is that our scoring system is arbitrary by necessity. Nevertheless, we tried to make the scoring system less subjective by taking the coefficients from the logistic regression equation as the basis for incremental changes in the score. Another limitation is that, due to small numbers, we cannot draw meaningful conclusions regarding the relationship between patient profiles, BP control, and the primary endpoint of the study. The main limitation of our study, however, is that it is a posthoc analysis of existing data, and that the concepts derived from this study need to be validated in other existing datasets from pivotal trials. Therefore, our conclusions cannot be extrapolated immediately to hypertension treatment. Nevertheless, our data could be taken as a starting point for analyzing other trials in the same way and to check whether other patient characteristics have added value for the patient profiles used. Although the profiles emerging from the present analysis can likely be applied to most hypertensive populations, it is conceivable that additional profiles may have to be included in the future.

When reproducible, patient profiles can help tailor treatment decisions for individual patients with hypertension. Nevertheless, a prospective randomized trial is necessary to explore whether patient profile-based treatment leads to better outcomes than guideline-based treatment.

## ACKNOWLEDGEMENTS

The authors wish to acknowledge Professor Talma Rosenthal and late professor Alain Castaigne for their contributions to the design and execution of the original INSIGHT trial.

This trial was executed and completed before clinical trial registries were instituted; therefore, no trial registration numbers were available.

Data from this study have been presented, in part, at the 34^th^ European Meeting on Hypertension and Cardiovascular Protection, Milan, 2025.

The original study was sponsored by Bayer AG. For the present analysis, no funding was obtained and none of the authors has any disclosures relevant to the present paper.

### Conflicts of interest

None with respect to the present study.

## Supplementary Material

Supplemental Digital Content

## Supplementary Material

Supplemental Digital Content

## Supplementary Material

Supplemental Digital Content
