# Supplementary material for: Patient-profiled treatment responses in a large hypertension trial: a posthoc analysis of the INSIGHT study
Source: J Hypertens. 2026 Mar 18;44(6):999–1004. doi: 10.1097/HJH.0000000000004284 (PMC13152050; doi:10.1097/HJH.0000000000004284)
Supplement: Supplemental Digital Content [file jhype-44-0999-s001.docx]

**Supplemental table 1. Summary of all possible profiles.**

| Profile # | Age (years) | SPB (mmHg) | Organ damage | Comorbidity | Prior treatment status |
| --- | --- | --- | --- | --- | --- |
| 1 | < 60 | <160 | No | None | Naïve |
| 2 | < 60 | <160 | No | None | Monotherapy |
| 3 | < 60 | <160 | No | Obesity | Naïve |
| 4 | < 60 | <160 | No | Obesity | Monotherapy |
| 5 | < 60 | <160 | No | Diabetes | Naïve |
| 6 | < 60 | <160 | No | Diabetes | Monotherapy |
| 7 | < 60 | <160 | No | Obesity and diabetes | Naïve |
| 8 | < 60 | <160 | No | Obesity and diabetes | Monotherapy |
| 9 | < 60 | <160 | Yes | None | Naïve |
| 10 | < 60 | <160 | Yes | None | Monotherapy |
| 11 | < 60 | <160 | Yes | Obesity | Naïve |
| 12 | < 60 | <160 | Yes | Obesity | Monotherapy |
| 13 | < 60 | <160 | Yes | Diabetes | Naïve |
| 14 | < 60 | <160 | Yes | Diabetes | Monotherapy |
| 15 | < 60 | <160 | Yes | Obesity and diabetes | Naïve |
| 16 | < 60 | <160 | Yes | Obesity and diabetes | Monotherapy |
| 17 | < 60 | >160 | No | None | Naïve |
| 18 | < 60 | >160 | No | None | Monotherapy |
| 19 | < 60 | >160 | No | Obesity | Naïve |
| 20 | < 60 | >160 | No | Obesity | Monotherapy |
| 21 | < 60 | >160 | No | Diabetes | Naïve |
| 22 | < 60 | >160 | No | Diabetes | Monotherapy |
| 23 | < 60 | >160 | No | Obesity and diabetes | Naïve |
| 24 | < 60 | >160 | No | Obesity and diabetes | Monotherapy |
| 25 | < 60 | >160 | Yes | None | Naïve |
| 26 | < 60 | >160 | Yes | None | Monotherapy |
| 27 | < 60 | >160 | Yes | Obesity | Naïve |
| 28 | < 60 | >160 | Yes | Obesity | Monotherapy |
| 29 | < 60 | >160 | Yes | Diabetes | Naïve |
| 30 | < 60 | >160 | Yes | Diabetes | Monotherapy |
| 31 | < 60 | >160 | Yes | Obesity and diabetes | Naïve |
| 32 | < 60 | >160 | Yes | Obesity and diabetes | Monotherapy |
| 33 | 60-80 | <160 | No | None | Naïve |
| 34 | 60-80 | <160 | No | None | Monotherapy |
| 35 | 60-80 | <160 | No | Obesity | Naïve |
| 36 | 60-80 | <160 | No | Obesity | Monotherapy |
| 37 | 60-80 | <160 | No | Diabetes | Naïve |
| 38 | 60-80 | <160 | No | Diabetes | Monotherapy |
| 39 | 60-80 | <160 | No | Obesity and diabetes | Naïve |
| 40 | 60-80 | <160 | No | Obesity and diabetes | Monotherapy |
| 41 | 60-80 | <160 | Yes | None | Naïve |
| 42 | 60-80 | <160 | Yes | None | Monotherapy |
| 43 | 60-80 | <160 | Yes | Obesity | Naïve |
| 44 | 60-80 | <160 | Yes | Obesity | Monotherapy |
| 45 | 60-80 | <160 | Yes | Diabetes | Naïve |
| 46 | 60-80 | <160 | Yes | Diabetes | Monotherapy |
| 47 | 60-80 | <160 | Yes | Obesity and diabetes | Naïve |
| 48 | 60-80 | <160 | Yes | Obesity and diabetes | Monotherapy |
| 49 | 60-80 | >160 | No | None | Naïve |
| 50 | 60-80 | >160 | No | None | Monotherapy |
| 51 | 60-80 | >160 | No | Obesity | Naïve |
| 52 | 60-80 | >160 | No | Obesity | Monotherapy |
| 53 | 60-80 | >160 | No | Diabetes | Naïve |
| 54 | 60-80 | >160 | No | Diabetes | Monotherapy |
| 55 | 60-80 | >160 | No | Obesity and diabetes | Naïve |
| 56 | 60-80 | >160 | No | Obesity and diabetes | Monotherapy |
| 57 | 60-80 | >160 | Yes | None | Naïve |
| 58 | 60-80 | >160 | Yes | None | Monotherapy |
| 59 | 60-80 | >160 | Yes | Obesity | Naïve |
| 60 | 60-80 | >160 | Yes | Obesity | Monotherapy |
| 61 | 60-80 | >160 | Yes | Diabetes | Naïve |
| 62 | 60-80 | >160 | Yes | Diabetes | Monotherapy |
| 63 | 60-80 | >160 | Yes | Obesity and diabetes | Naïve |
| 64 | 60-80 | >160 | Yes | Obesity and diabetes | Monotherapy |
