# Supplementary material for: Patient-profiled treatment responses in a large hypertension trial: a posthoc analysis of the INSIGHT study
Source: J Hypertens. 2026 Mar 18;44(6):999–1004. doi: 10.1097/HJH.0000000000004284 (PMC13152050; doi:10.1097/HJH.0000000000004284)
Supplement: Supplemental Digital Content [file jhype-44-0999-s002.docx]

**Supplemental file 2. Development of the model to calculate risk scores.**

For the calculation of the risk score, we used the four predictor categories: age, severity, comorbidity and treatment status as mentioned in table 1 of the main manuscript.

In general, the multivariable logistic regression equation which describes the relationship between an outcome variable and four predictor categories is:

Outcome = constant + ß1*cat1 + ß2*cat2 + ß3*cat3 + ß4*cat4.

For the present analysis, outcome is the probability of treatment failure at six months and ß1 to ß4 are the regression coefficients. However, three of the four categories have more than two options. In these cases, the most favorable option (from a clinical point of view) was chosen as the reference category and omitted from the equation. We then created dummy binary variables (0/1) for the remaining options. This led to the following regression coefficients:

Co-amilozide Nifedipine

Cat1 (age >60 vs age<60):  ß1 = 1.12 ß1= 1.28

Cat2 (SBP<160 mmHg. with organ damage): ß2a = 0.70 ß2a = 0.86 (SBP>160 mmHg, no organ damage): ß2b = 2.67 ß2b = 1.64

(SBP>160 mmHg with organ damage): ß2c = 3.16 ß2c = 2.01

Cat3 (obesity): ß3a = 1.26 ß3a = 1.04 (diabetes): ß3b = 1.74 ß3b = 1.14

(obesity plus diabetes): ß3c = 1.50 ß3c = 1.56

Cat4 (previously treated vs no treatment): ß4= 1.44 ß4 = 1.66

The constant in the regression equation is: 0.17 0.30

For each category, the product of the regression coefficient and the corresponding categorical variable was taken as the risk score for that category. The sum of the constant and the risk scores determined the total risk score for a profile.
