# Supplementary material for: Patient-profiled treatment responses in a large hypertension trial: a posthoc analysis of the INSIGHT study
Source: J Hypertens. 2026 Mar 18;44(6):999–1004. doi: 10.1097/HJH.0000000000004284 (PMC13152050; doi:10.1097/HJH.0000000000004284)
Supplement: Supplemental Digital Content [file jhype-44-0999-s003.docx]

**Supplemental table 3. Summary of all profiles in the INSIGHT study.**

| Profile number | N | | Age (years) | SPB (mmHg) | Organ damage | Comorbidity | Treatment status |
| --- | --- | --- | --- | --- | --- | --- | --- |
|  | Co-amilozide | Nifedipine |  |  |  |  |  |
| 1 | 15 | 21 | < 60 | <160 | No | None | Naïve |
| **2** | **58** | **74** | **< 60** | **<160** | **No** | **None** | **Previously treated** |
| 4 | 7 | 7 | < 60 | <160 | No | Obesity | Naïve |
| 5 | 28 | 21 | < 60 | <160 | No | Obesity | Previously treated |
| 8 | 9 | 9 | < 60 | <160 | No | Diabetes | Previously treated |
| 10 | 0 | 3 | < 60 | <160 | No | Obesity and diabetes | Naïve |
| 11 | 5 | 13 | < 60 | <160 | No | Obesity and diabetes | Previously treated |
| 13 | 3 | 2 | < 60 | <160 | Yes | None | Naïve |
| 14 | 24 | 25 | < 60 | <160 | Yes | None | Previously treated |
| 16 | 1 | 1 | < 60 | <160 | Yes | Obesity | Naïve |
| 17 | 9 | 4 | < 60 | <160 | Yes | Obesity | Previously treated |
| 20 | 3 | 3 | < 60 | <160 | Yes | Diabetes | Previously treated |
| 23 | 2 | 2 | < 60 | <160 | Yes | Obesity and diabetes | Previously treated |
| 25 | 30 | 31 | < 60 | >160 | No | None | Naïve |
| **26** | **144** | **147** | **< 60** | **>160** | **No** | **None** | **Previously treated** |
| 28 | 17 | 13 | < 60 | >160 | No | Obesity | Naïve |
| **29** | **79** | **77** | **< 60** | **>160** | **No** | **Obesity** | **Previously treated** |
| 31 | 2 | 1 | < 60 | >160 | No | Diabetes | Naïve |
| 32 | 27 | 30 | < 60 | >160 | No | Diabetes | Previously treated |
| 34 | 2 | 2 | < 60 | >160 | No | Obesity and diabetes | Naïve |
| 35 | 35 | 39 | < 60 | >160 | No | Obesity and diabetes | Previously treated |
| 37 | 12 | 19 | < 60 | >160 | Yes | None | Naïve |
| **38** | **80** | **83** | **< 60** | **>160** | **Yes** | **None** | **Previously treated** |
| 40 | 1 | 1 | < 60 | >160 | Yes | Obesity | Naïve |
| 41 | 38 | 43 | < 60 | >160 | Yes | Obesity | Previously treated |
| 43 | 1 | 1 | < 60 | >160 | Yes | Diabetes | Naïve |
| 44 | 15 | 10 | < 60 | >160 | Yes | Diabetes | Previously treated |
| 47 | 19 | 14 | < 60 | >160 | Yes | Obesity and diabetes | Previously treated |
| 49 | 16 | 18 | 60-80 | <160 | No | None | Naïve |
| **50** | **70** | **82** | **60-80** | **<160** | **No** | **None** | **Previously treated** |
| 52 | 8 | 3 | 60-80 | <160 | No | Obesity | Naïve |
| 53 | 39 | 43 | 60-80 | <160 | No | Obesity | Previously treated |
| 55 | 1 | 4 | 60-80 | <160 | No | Diabetes | Naïve |
| 56 | 16 | 17 | 60-80 | <160 | No | Diabetes | Previously treated |
| 58 | 2 | 0 | 60-80 | <160 | No | Obesity and diabetes | Naïve |
| 59 | 12 | 19 | 60-80 | <160 | No | Obesity and diabetes | Previously treated |
| 61 | 10 | 14 | 60-80 | <160 | Yes | None | Naïve |
| **62** | **88** | **87** | **60-80** | **<160** | **Yes** | **None** | **Previously treated** |
| 64 | 5 | 1 | 60-80 | <160 | Yes | Obesity | Naïve |
| 65 | 21 | 19 | 60-80 | <160 | Yes | Obesity | Previously treated |
| 68 | 8 | 6 | 60-80 | <160 | Yes | Diabetes | Previously treated |
| 70 | 1 | 0 | 60-80 | <160 | Yes | Obesity and diabetes | Naïve |
| 71 | 3 | 3 | 60-80 | <160 | Yes | Obesity and diabetes | Previously treated |
| **73** | **68** | **60** | **60-80** | **>160** | **No** | **None** | **Naïve** |
| **74** | **355** | **367** | **60-80** | **>160** | **No** | **None** | **Previously treated** |
| 76 | 30 | 25 | 60-80 | >160 | No | Obesity | Naïve |
| **77** | **205** | **184** | **60-80** | **>160** | **No** | **Obesity** | **Previously treated** |
| 79 | 13 | 9 | 60-80 | >160 | No | Diabetes | Naïve |
| **80** | **100** | **95** | **60-80** | **>160** | **No** | **Diabetes** | **Previously treated** |
| 82 | 9 | 12 | 60-80 | >160 | No | Obesity and diabetes | Naïve |
| **83** | **100** | **78** | **60-80** | **>160** | **No** | **Obesity and diabetes** | **Previously treated** |
| **85** | **93** | **97** | **60-80** | **>160** | **Yes** | **None** | **Naïve** |
| **86** | **660** | **593** | **60-80** | **>160** | **Yes** | **None** | **Previously treated** |
| 88 | 17 | 10 | 60-80 | >160 | Yes | Obesity | Naïve |
| **89** | **119** | **151** | **60-80** | **>160** | **Yes** | **Obesity** | **Previously treated** |
| 91 | 14 | 8 | 60-80 | >160 | Yes | Diabetes | Naïve |
| **92** | **138** | **148** | **60-80** | **>160** | **Yes** | **Diabetes** | **Previously treated** |
| 94 | 5 | 3 | 60-80 | >160 | Yes | Obesity and diabetes | Naïve |
| **95** | **61** | **68** | **60-80** | **>160** | **Yes** | **Obesity and diabetes** | **Previously treated** |

SBP: systolic blood pressure. Profiles in bold indicate the ones used for analysis.
